# Detecting rare asymmetrically methylated cytosines and decoding methylation patterns in the honeybee genome

**DOI:** 10.1098/rsos.170248

**Published:** 2017-09-06

**Authors:** Laura Welsh, Ryszard Maleszka, Sylvain Foret

**Affiliations:** Research School of Biology, The Australian National University, Canberra, Australian Capital Territory 2601, Australia

**Keywords:** DNA methylation, epigenomics, social insect

## Abstract

Context-dependent gene expression in eukaryotes is controlled by several mechanisms including cytosine methylation that primarily occurs in the CG dinucleotides (CpGs). However, less frequent non-CpG asymmetric methylation has been found in various cell types, such as mammalian neurons, and recent results suggest that these sites can repress transcription independently of CpG contexts. In addition, an emerging view is that CpG hemimethylation may arise not only from deregulation of cellular processes but also be a standard feature of the methylome. Here, we have applied a novel approach to examine whether asymmetric CpG methylation is present in a sparsely methylated genome of the honeybee, a social insect with a high level of epigenetically driven phenotypic plasticity. By combining strand-specific ultra-deep amplicon sequencing of illustrator genes with whole-genome methylomics and bioinformatics, we show that rare asymmetrically methylated CpGs can be unambiguously detected in the honeybee genome. Additionally, we confirm differential methylation between two phenotypically and reproductively distinct castes, queens and workers, and offer new insight into the heterogeneity of brain methylation patterns. In particular, we challenge the assumption that symmetrical methylation levels reflect symmetry in the underlying methylation patterns and conclude that hemimethylation may occur more frequently than indicated by methylation levels. Finally, we question the validity of a prior study in which most of cytosine methylation in this species was reported to be asymmetric.

## 1. Introduction

Methylation of cytosines in DNA is part of the epigenetic communication system that controls gene expression in most eukaryotic species [1]. It has been implicated in various biological processes, including development, environmental responses and brain plasticity [2,3]. Cytosine methylation occurs predominantly in the CG dinucleotides (or CpGs) where complementary base pairing of cytosine and guanine provides the sequence context for ‘symmetric’ methylation on both strands. Non-CpG methylation at CH and CHH sites, referred to as ‘asymmetric’ is less frequent in animals and has been reported only in certain situations, notably in mammalian cell types. For example, this type of methylation accumulates in neurons, but not glia, during mammalian brain development [4] and gain/loss of asymmetric non-CpG methylation in the CpA context has been detected during male germ-cell differentiation [5]. Although the role of asymmetric cytosine methylation remains debatable, recent results support the idea that methylated CpH sites have an intrinsic capacity to repress transcription independently of CpG contexts [6].

While the framework for methylation of both strands exists it is not entirely clear whether this process is consistent and always completed. Indeed, CpG hemimethylation could act as a stable marker, potentially conveying a different functional output to double-stranded/symmetrical methylation. In vertebrates, several studies have suggested that CpGs are not always methylated on both strands [7–9]. For example, Shao *et al.* [10] identified hemimethylation in human cancerous cells and healthy, control cells, suggesting hemimethylation may arise when cellular processes are deregulated but may also be a normal feature of the methylome.

Until recently, investigation of asymmetrical CpG methylation has been limited by a lack of data and tools to capture the methylation landscape. Past studies of methylation have explored methylation along a single strand or by combining bisulfite sequencing data from both strands, such that any differences between strands are obscured [11,12]. Studying asymmetry throughout the genome requires strand-specific bisulfite sequencing data of sufficient coverage, which remains expensive. Additionally, the dominant approach to characterizing patterns of methylation has been to perform bisulfite sequencing on a mixture of DNA originating from different individuals or cells in a tissue, potentially with different methylation patterns, to calculate the mean methylation level at each CpG; [13]. This approach loses the methylation profile of the original DNA fragment and information about the surrounding CpGs [14]. As methylation levels average information from all reads, all or nearly all of the fragments of DNA in the bisulfite sequencing experiment must be hemimethylated in the same direction for hemimethylation to be detected. Recently, tools such as Methpat and MPFE have been developed to study methylation patterns rather the levels [14]. Amplicon bisulfite sequencing enables high coverage sequence data to be generated for both strands of DNA, allowing for in-depth analysis of methylation patterns and comparison of methylation between strands at specific loci [15–18].

The honeybee in which phenotypic polymorphism and certain types of behaviour are driven by DNA methylation is an important model for methylomics [19–21]. In contrast to heavily methylated mammalian and plant genomes, insect genomes are only sparsely methylated with most of methyl-cytosines found in gene bodies and often near splice sites. Until recently, no significant asymmetric methylation was detected in the honeybee methylomes suggesting that this type of DNA modification is very rare in this insect and possibly in most invertebrates [12,22]. In this context, a recent report by Niazi *et al.* [23] claiming that in the honeybee, much of the methylation is asymmetric is highly surprising. Here, we reinvestigate this unanticipated result by applying a novel combination of *in silico* analysis and deep strand-specific amplicon bisulfite sequencing that allow exploring both methylation levels and methylation patterns to identify consistent cases of hemimethylation, and to assess symmetry of methylation patterns.

This study has revealed very little difference in the methylation levels between strands with the exception of a small number of consistently hemimethylated sites. In particular, one CpG site was found to be consistently asymmetrical across phenotypic morphs and datasets. Analysis of three loci shows asymmetrical patterns suggesting that methylation levels mask cryptic asymmetry in the underlying methylation patterns. In accord with our previous report, these new data confirm that in the brain of newly emerged honeybees methylation patterns are diverse and potentially caste-specific. Our findings also suggest that claims of asymmetric CpG methylation ‘prevalence’ in other insects [24] need to be reinvestigated with more sensitive technologies.

Our study is the first to identify consistently hemimethylated sites in the honeybee and apply the methylation pattern approach to exploring strand asymmetry and inter-caste differences. We use the term asymmetrical methylation to refer to differences in methylation levels or patterns in a DNA sample that are underpinned by hemimethylation of individual DNA fragments. In the context of methylation levels, we define ‘asymmetry’ as a statistically significant difference in methylation levels between strands. Our findings raise new questions about the origin and role of hemimethylation. Practically, this study validates the use of amplicon bisulfite sequencing and the methylation patterns approach for studying methylomes and exploring methylation profiles to understand how methylation patterns are established and the instructions they encode.

## 2. Material and methods

### 2.1. Bioinformatics analysis of whole-genome data

Whole-genome bisulfite sequencing data for newly emerged queen (Q), nurse (N) and three-week old forager (F) brains, obtained from the study by Herb *et al.* [21], were used for genome-wide analysis of asymmetry. Nurses and foragers refer to two types of functionally distinct worker bees performing either indoor (N) or outdoor (F) tasks. Data from pooled replicates were combined within castes to generate a single, higher coverage dataset for each caste. Sequencing reads were mapped to the honeybee Amel 4.5 reference genome using Bismark with Bowtie2, a short read mapper [25,26]. WGBS reads were trimmed by five base pairs at each end. The numbers of methylated and unmethylated reads were tested against a binomial distribution and *p*-values were adjusted for multiple testing using the Benjamini–Hochberg method [27]. A 1% non-conversion rate was used and methylation calls were made on the combined reads from both strands. CpGs with less than 10 reads on each strand or total coverage above the 95th percentile were excluded.

Methylation levels were computed for each CpG on each strand, using the formula *b* = *M*/*M* + *U*, where *M* is the number of methylated reads at a site and *U* the number of unmethylated reads. The correlation between methylation levels on the plus strand and minus strand was assessed using Pearson's correlation and data were plotted using the ggplot2 R package.

Individual CpG sites were tested for asymmetry using Fisher's exact test on the number of methylated and unmethylated reads on each strand and were adjusted using a Benjamini–Hochberg correction. A sliding window approach was used to test for asymmetrically methylated regions or clusters of CpGs. A window size of 1800 bp containing at least eight methylated CpGs was used and paired *t*-tests were performed on the methylation levels at each CpG on each strand. The *P*-values were corrected as above.

Whole-genome bisulfite sequencing data accession: NCBI SRA, SRA050798. Amplicon bisulfite sequencing data: the Dryad repository (http://dx.doi.org/10.5061/dryad.7nb8q) [28].

### 2.2. Investigation of the false positive rate

To determine whether the number of asymmetrically methylated CpGs detected was greater than what we would expect to find if there was no asymmetry, the reads at each CpG were shuffled so that methylated and unmethylated reads were randomly assigned to the plus or minus strand. This was repeated for each methylated CpG, 100 times. After shuffling, Fisher's exact test and paired *t*-tests were used to identify asymmetrical CpGs and regions in the shuffled datasets.

### 2.3. Amplicon bisulfite sequencing

Amplicon bisulfite sequencing [15,17] was carried out for selected asymmetrical CpG sites identified in whole-genome data and two illustrator genes *cabin-1*, *nadrin-2* in newly emerged queen and worker brains. Both *cabin-1* and *nadrin-2* were found previously to be differentially methylated in brains of queens and workers dissected from mature older individuals [22], but not in larval heads [12]. Both genes have more methylated CpGs than most of the honeybee genes (which are only moderately methylated) and thus, are more useful for the analyses reported in our manuscript. Importantly, we did not find any sequence variants in the gene regions selected for both amplicons, which could potentially obscure our findings. Bees were obtained from our hives located on campus and processed as described previously [16,29–31]. Brains were dissected in 0.1xNaCl-Tris-EDTA buffer under a dissecting microscope [16]. The hypopharyngeal and salivary glands were removed from the brain due to their extremely high expression of certain genes and different methylation profile [19]. Brains were pooled in three sets of five brains for queens and workers. Brain DNA was extracted using Epicentre's MasterPure kit. Following extraction, DNA underwent bisulfite treatment using the Qiagen Bisulfite kit. A nested PCR design and custom primers were used to amplify bisulfite-treated DNA. Amplicons were amplified in duplicate at the nested step to increase the final DNA yield. The total reaction volume was run on agarose gel (1.5%, using ethidium bromide or gel red dye) to distinguish DNA bands. The band of desired amplicon length was excised from the gel using a scalpel over a UV box. DNA was purified from the gel using Zymogen Gel DNA Recovery kit.

#### 2.3.1. Library preparation and sequencing

Amplicon DNA concentrations were measured using a high sensitivity GX Labchip and combined in equimolar amounts. Libraries were then prepared from the pooled DNA using the NEB Next UltraTM Directional RNA Library Prep kit with slight modifications.

Primers with unique indices were used and 13 cycles of PCR were performed using the recommended cycling profile. PCR products were purified using AMPure XP beads and DNA was eluted in 40 µl of nuclease-free water. The DNA concentration of library was quantified using a high sensitivity GX Labchip and libraries were pooled in equimolar amounts. Pooled libraries underwent a final AMPure XP bead purification step to remove remaining adaptors. Illumina Miseq sequencing was carried out by the Biomolecular Resource Facility at the John Curtin School of Medical Research at the Australian National University. See our previous studies, for more details [15,17].

### 2.4. Analysis of amplicon data

Sequencing reads were trimmed and mapped to a reference containing the amplicon sequences using Bismark. Methylation data were extracted by Bismark using the ‘non-directional’ option and methylation calls were corrected using a 1% non-conversion rate. To test for asymmetry, a linear mixed model was fitted to model methylation level as a function of strand and sample, using the R package lme4.

### 2.5. Pattern analysis

To identify methylation patterns, Bismark output of *cabin-1*, *nadrin-2* and QNFC1, was parsed to Methpat, a program that uses methylation data to count the methylation patterns present [14]. Methpat outputs methylation patterns as a string of zeros and ones, corresponding to unmethylated and methylated CpGs along a read. Patterns containing spurious/phantom CpG sites and truncated patterns were filtered, such that remaining patterns were of equal length and overlapped the same CpG sites. Patterns with abundance below 1% of the total coverage across the amplicon were removed.

MPFE was then used to estimate pattern frequencies on each strand. Multinomial logistic regression was used to test for differences in the proportions of different patterns on each strand and for differences in patterns between replicates, using the R package ‘nnet’. We modelled the probability of observing a particular pattern as a function of strand and replicate. Models with and without strand as an explanatory variable were compared to examine the overall contribution of strand to the model fit. Finally, patterns present in queen and worker samples were compared and tested using multinomial logistic regression, modelling the estimated frequency of each pattern as a function of strand, replicate and caste. To estimate the variation in the difference in pattern frequency between strands, reads were resampled 1000 times to determine confidence intervals.

## 3. Results

### 3.1. Characterization of methylation asymmetry using methylation levels approach

#### 3.1.1. Symmetry of genome-wide methylation levels

**Figure 1.**
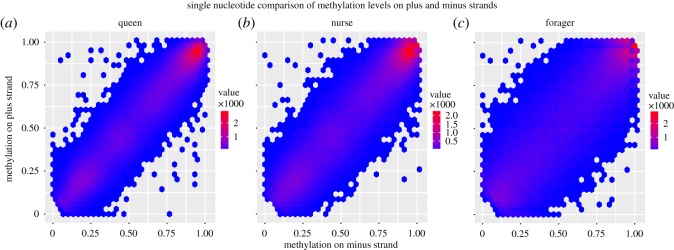
Comparison of methylation levels in (*a*) queen, (*b*) nurse and (*c*) forager datasets. Methylation levels were strongly correlated in each functional category, with the majority of points showing symmetrical methylation levels and a small proportion of asymmetrical sites (*R*_queen_^2^ = 0.93, *R*_forager_^2^ = 0.84, *R*_worker_^2^ = 0.90, Pearson's correlation).

Strand-specific methylation levels were explored throughout the brain methylomes in queens (Q) and two functionally distinct worker bees, nurses (N) and foragers (F). A strong positive correlation was observed between methylation levels on each strand for all castes (*R*^2 ^= 0.93 in queen dataset, 0.84 in forager and 0.90 in worker). The majority of sites were found to be methylated symmetrically with few asymmetrically methylated sites in each caste (figure 1).

#### 3.1.2. Asymmetrically methylated CpGs

A small number of asymmetrically methylated sites were detected in each type of bees using Fisher's exact test on the proportion of methylated and unmethylated reads at each CpG; 38 asymmetrically methylated CpGs were identified in the queen dataset, while eight and 10 sites were found to be asymmetrically methylated in the nurse and forager datasets, respectively (table 1). In all three groups, asymmetric CpGs correspond to less than 0.05% of all methylated CpGs. Asymmetrically methylated CpGs are located throughout the genome, with the majority situated within genes (table 1). A single site was found to be asymmetrical in all datasets and additionally, five CpG sites were found to be asymmetrical in both the queen and nurse datasets (electronic supplementary material, figure S1).

**Table 1.** Asymmetrical CpG sites identified using Fisher's exact test. Thirty-eight sites were found to be asymmetrical in queens, with 8 and 10 sites detected in nurses and foragers. Asymmetrical CpGs corresponded to less than 0.05% of all methylated CpGs studied. Of the sites identified as asymmetrical, only one was found to have difference in methylation levels above 0.9.

	queen	nurse	forager
number of asymmetrical sites	38	8	10
% of all sites tested	0.04	0.01	0.01
number of sites situated within known genes (exons)	29	6 (5)	10 (9)
number of sites with difference in methylation levels greater than 0.9	0	1	1

The false positive rate associated with detecting asymmetrically methylated CpGs and regions was estimated by shuffling reads to generate ‘symmetrical’ datasets and testing for asymmetry. One or no asymmetrically methylated CpGs was identified in a small number of shuffled forager and queen datasets using Fisher's exact test (table 2). No asymmetry was identified in the shuffled nurse datasets. Despite the number of asymmetrical sites detected in our data being very small in all types of bees, more sites were detected than we would expect if there was no asymmetry.

**Table 2.** Investigation of false positive asymmetrically methylated CpGs in shuffled datasets. 100 symmetrical datasets were generated for each caste by shuffling and randomly assigning reads to each strand. For each dataset, Fisher's exact test was carried out for each CpG site.

	queen	nurse	forager
number of shuffled datasets containing asymmetrically methylated CpGs	1	0	2
number of asymmetrical CpGs in shuffled datasets	1	0	1
number of asymmetrical CpGs in study dataset	38	8	10

To increase the power of our analyses, deep coverage amplicon bisulfite sequencing data were generated for five asymmetrically methylated CpGs identified in the whole-genome bisulfite sequencing datasets. A linear mixed model was fitted to test for differences between strands at asymmetrical sites. Three of the five CpG sites tested were found to be asymmetrically methylated, consistent with whole-genome data (*p* < 0.5, linear mixed model; table 3). At NC8 and QC21, however, the difference in methylation levels between strands, averaged across replicates, was lower than in whole-genome datasets. Both sites were found to occur within genes. QC20 is located in an exon but was not associated with alternatively spliced transcript, while WC8 situated within an intron.

**Table 3.** Comparison of whole-genome and amplicon bisulfite sequencing data at asymmetrically methylated CpGs. Functional category designates whether the site was asymmetrical in queen (Q), nurse (N) or forager (F) whole-genome data. The difference in methylation level (plus–minus strand) was computed for each site in whole-genome data and amplicon data (mean of replicates was calculated). Asymmetry was tested using a linear mixed model, CpGs are reported as asymmetrically methylated when *p* < 0.05.

CpG ID	functional category	gene ID	mean difference (whole genome)	asymmetry in amplicon data	mean difference (amplicon)
QNFC1	Q,N,F	GB51130	0.90	yes	0.87
QNC2	Q,N	GB48544	0.57	no	0.05
NC8	N	GB44288	0.74	yes	0.20
QC20	Q	GB55279	−0.77	no	−0.26
QC21	Q	GB42155	−0.62	yes	−0.32

**Figure 2.**
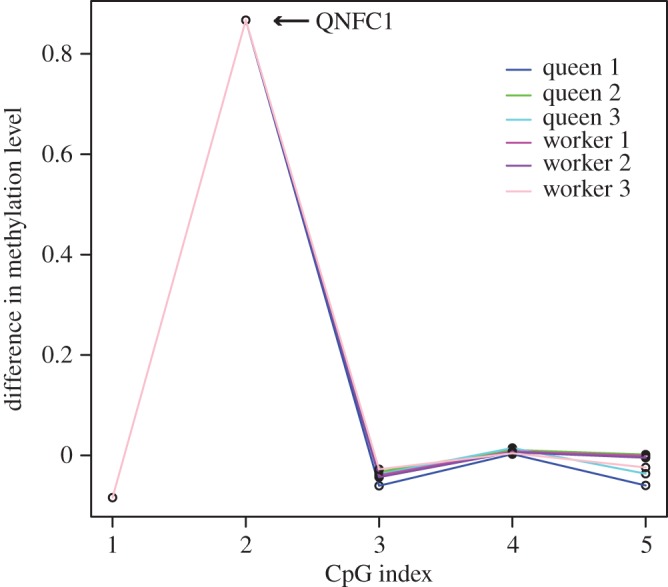
Difference in methylation levels at QNFC1 and surrounding CpGs using deep amplicon sequencing. Amplicon data from three queen and three worker replicates for the region including QNFC1, an asymmetrically methylated site according to whole-genome data for all castes. The difference between methylation levels on each strand was highly conserved between replicates. In figures 2–7, CpG index refers to the position and order of CpGs within the amplicon and not a consecutive string of CpGs.

The CpG site found to be asymmetrical and hemimethylated in queen, nurse and forager datasets, referred to as QNFC1, is asymmetrically methylated in amplicon data for queens and nurses and across all biological replicates (figure 2). The difference in methylation levels within the region was highly conserved across replicates, with all replicates showing a difference of approximately 0.87 between strands at the site QNFC1 (figure 2). Further investigation of this site revealed that QNFC1 is situated within an exon of GB51130, a gene predicted to encode E3 ubiquitin-protein ligase. Visualization of RNAseq data from newly emerged workers (no functional specialization) in IGV reveals that GB51130 has an alternatively spliced transcript and that QNFC1 is located six bases downstream from a splice site (electronic supplementary material, figure S2).

#### 3.1.3. Asymmetrically methylated regions

**Figure 3.**
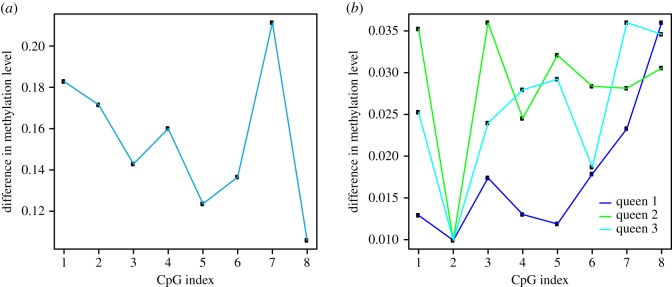
(*a*) Methylation profile of asymmetrically methylated region in queen dataset (QR10). Graph of the difference in methylation levels between strands. Methylation levels were similar on each strand at 8 CpG sites within QR10. Despite a significant difference between methylation levels on each strand, the magnitude of the difference was small, ranging between 0.10 and 0.22 (paired *t*-test, *p* < 0.5). (*b*) Difference in methylation levels at QR10. The difference in methylation levels at each CpG ranged between 1 and 4%.

Small regions of the genome were tested for differences in methylation levels between strands to determine whether regions of asymmetrical methylation occurred, using a sliding window approach. Two asymmetrical regions were identified in the whole-genome queen dataset, while in the forager and nurse, only one region was identified (paired *t*-test, *p* < 0.05). There was no overlap in the regions detected in each group (electronic supplementary material, figure S3). Asymmetrically methylated CpG sites are not situated within the identified asymmetrical regions and the difference in methylation levels between the two strands is small, ranging from 0 to 22% (figure 3*a*).

Investigation of false positive regions identified a small number of asymmetrical regions in shuffled datasets (table 4). One asymmetrically methylated region was identified in queen and forager shuffled datasets, while no asymmetric regions were detected in shuffled nurse datasets.

**Table 4.** Investigation of false positive asymmetrically methylated regions in shuffled datasets. 100 symmetrical datasets were generated for each caste by shuffling and randomly assigning reads to each strand. For each dataset, a sliding window analysis with paired *t*-test was used to identify asymmetrically methylated regions.

	queen	nurse	forager
number of shuffled datasets containing asymmetrically methylated regions	1	0	2
number asymmetrical regions in shuffled datasets	1	0	1
number of asymmetrical regions in study dataset	2	1	1

Amplicon bisulfite sequencing was performed to verify asymmetrical methylation levels observed at the region referred to as ‘QR10’ in the queen whole-genome bisulfite sequencing dataset. The difference in methylation levels between strands at CpGs in QR10 was found to be very small, ranging from 1 to 4% (figure 3*b*). The difference in methylation levels was therefore less pronounced than observed at QR10 in whole-genome data, where levels differed by up to 22% (figure 3*a*). This is probably due to the difference in coverage between the two datasets, as the amplicon bisulfite sequencing data had more than 100 times greater coverage than the whole-genome bisulfite sequencing dataset.

### 3.2. Methylation patterns reveal cryptic asymmetry

**Figure 4.**
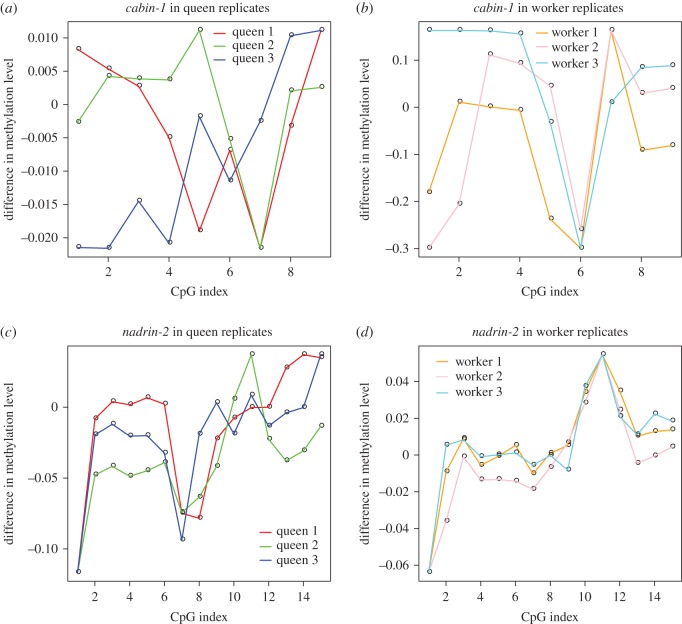
Methylation levels from amplicon bisulfite sequencing data at CpG sites within *cabin-1* and *nadrin-2* in queen and worker samples.

Analysis of methylation patterns in amplicons within two genes *cabin-1* and *nadrin-2* shows that, despite little difference in methylation levels between strands, methylation patterns were not symmetric. CpG sites in *nadrin-2* showed up to 10% difference in methylation levels between strands from amplicon sequencing data (figure 4*c,d*). In *cabin-1*, the difference in methylation levels between strands varied from 1 to 30% (figure 4*a,b*). All CpG sites were found to be asymmetrically methylated using a *χ*^2^-test (*p* < 0.5); however, the difference between strands was very small.

**Figure 5.**
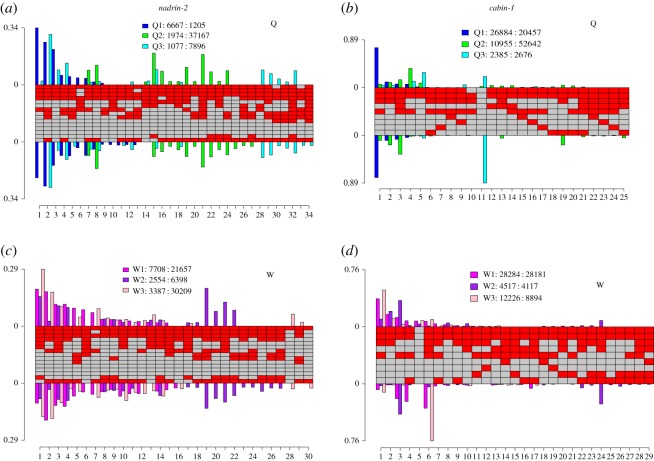
Methylation patterns in *nadrin-2* (*a,c*) and *cabin-1* (*b,d*) in newly emerged queens (Q) and workers (W). The central panel illustrates the methylation patterns observed, with each column depicting a different methylation pattern, with red squares corresponding to methylated CpGs and grey squares to unmethylated CpGs. Barplots illustrate the estimated frequency of each pattern on the plus strand (above) and minus strand (below) for each replicate. The legend specifies the coverage on each strand, shown as Sample name: Coverage on the plus strand: Coverage on the minus strand.

By contrast, methylation patterns were found to have different frequencies on each strand in *cabin-1* and *nadrin-2* amplicons (figure 5). In *nadrin-2*, the overall distribution of patterns across strands appeared even; however, the frequencies of 29 out of 34 patterns in queens and 23 out of 30 patterns in workers were significantly different between strands (multinomial logistic regression, *p* < 0.05; figure 5*a,c*). In *cabin-1*, methylation patterns were visibly uneven across strands (figure 5*b,d*). While the original pairing of patterns on individual DNA fragments could not be deduced, the observed differences in pattern frequencies on each strand imply that some DNA fragments have a different methylation pattern on each strand. In sample Q3, for example, pattern 11 (completely unmethylated) was highly abundant on the bottom strand and very rare on the top strand, suggesting that asymmetrical pairing occurs between methylated patterns on the top strand and an unmethylated pattern on the bottom strand. Similarly, in sample W3, the bottom strand was dominated by pattern 6, which was rare on the top strand (figure 5). In total, 28 out of the 29 patterns observed in worker replicates and 23 out of the 25 patterns in queens had significantly different frequencies on each strand (multinomial logistic regression, *p* < 0.05). Different methylation patterns on each strand suggest that one or more of the CpG sites were hemimethylatedon the original fragment.

Methylation patterns were resampled to estimate the difference in pattern frequencies between strands for *cabin-1* and *nadrin-2* (electronic supplementary material, figures S4, S5)*.* Bootstrapping revealed that the difference between strands for patterns in *cabin-1* ranged from 0 to 30%, and 0 to 10% in *nadrin-2*. In *cabin-1*, there was little variation in the effect size between resampling events (electronic supplementary material, figure S4).

**Figure 6.**
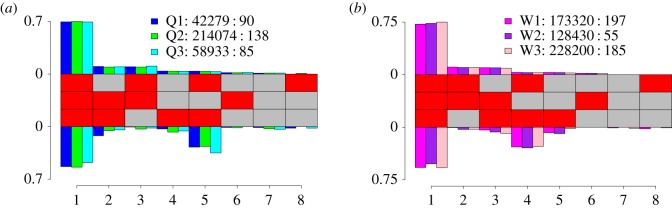
Methylation patterns in amplicon containing QNFC1 in newly emerged queens (*a*) and workers (*b*). The central panel illustrates the methylation patterns observed, with each column depicting a different methylation pattern, with red squares corresponding to methylated CpGs and grey squares to unmethylated CpGs. The middle CpG is QNFC1, the asymmetrical site detected in queens, foragers and workers (see §4.1 and figure 2). Barplots illustrate the estimated frequency of each pattern on the plus strand (above) and minus strand (below) for each replicate. The legend specifies the coverage on each strand, shown as Sample name: Coverage on the plus strand: Coverage on the minus strand.

Amplicon bisulfite sequencing of the region surrounding QNFC1, the consistently hemimethylated site in queen, nurse and forager whole-genome datasets, was performed to characterize methylation patterns. Eight methylation patterns were observed at QNFC1 and flanking CpGs (figure 6). All patterns in worker replicates and five out of eight patterns in queens were found to have different estimated frequencies on each strand, differing by up to 0.30 (multinomial regression, *p* < 0.05). The greatest difference in pattern frequency between strands was observed at pattern 5 in queen replicates and pattern 4 in worker replicates (figure 6). This pattern was unmethylated at QNFC1 and was more abundant on the minus strand. The low frequency of this pattern on the plus strand suggests that this pattern may pair with a different pattern on the plus strand which may be methylated at QNFC1. This supports previous findings of hemimethylation of the plus strand at QNFC1. Patterns 2–8 lacked methylation at one or more CpGs, suggesting that hemimethylation may also occur at the sites flanking QNFC1 (figure 6).

Comparing the residual deviances of the model incorporating strand with a model without strand, we found there was a large difference (greater than 1000) in the residual deviances for QNFC1, *cabin-1* and *nadrin-2*. This suggests that strand improves the fit of the model, and corroborates the differences suggested by *p*-values.

### 3.3. Methylation patterns offer new insight into the honeybee brain methylome

#### 3.3.1. Diversity of methylation patterns

The methylation pattern approach provides novel information about the newly emerged queen and worker brain methylomes. In the amplicons studied, a heterogeneous mix of methylation patterns was identified within replicates. Both *nadrin-2* and *cabin-1* were found to have a dominant methylation pattern, with multiple minor patterns. In *nadrin-2*, for example, the dominant pattern represented up to 30% of all patterns (figure 5*a,c*). By contrast, the methylation profile of *cabin-1* was characterized by a dominant pattern in each replicate, contributing up to 80% of the total abundance alongside rare patterns, with the exception of Q2 and W1 (figure 5*b,d*).

In addition, methylation patterns were found to vary between replicates within a caste. For example, in queen replicates, the dominant patterns in *nadrin-2* for Q1 and Q3 were not present in Q2, which had a unique set of patterns (figure 5). To explore the impact of replicate on our results, we compared the residual deviances of the model incorporating replicate with a model without replicate and found there to be a large difference (greater than 70 000) in the residual deviances for QNFC1, *cabin-1* and *nadrin-2*. This suggests that replicate explains some of the observed variation in methylation pattern frequencies.

In *nadrin-2* and *cabin-1*, approximately 30 different methylation patterns were observed (figure 5). This corresponds to a small fraction of all possible patterns (the total number of possible patterns in a locus with *n* CpG sites is 2*^n^*). In *nadrin-2*, for example, we observed 30 out of 32 768 (2^15^) possible patterns. Three CpGs were found to be consistently unmethylated in patterns in queens and workers, suggesting a fixed methylation status. This may be due to single-nucleotide polymorphisms altering the CpG sequence, preventing methylation [32]. The remaining CpGs were variably methylated or unmethylated (figure 5), which may be due to stochasticity of methylation mechanisms [33].

#### 3.3.2. Differences in methylation patterns in queens and workers

Estimated methylation pattern frequencies were compared for newly emerged queen and worker brains. Multinomial logistic regression indicates that a large percentage of the patterns observed had significantly different frequencies in queens and workers (100% of patterns in QNFC1 amplicon, 91% in *nadrin-2* and 64% in *cabin-1*, *p* < 0.05).

Plotting the mean pattern frequency of all replicates within a group revealed that all patterns at QNFC1 were present in both castes (queens and workers), but showed slight differences in the frequency on each strand (electronic supplementary material, figure S6).

**Figure 7.**
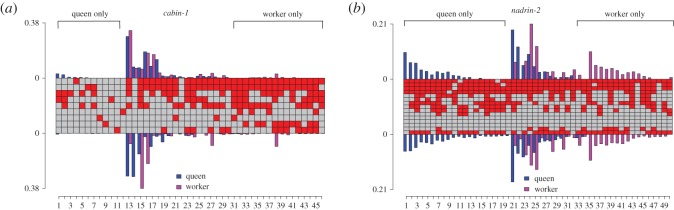
Comparison of patterns in *cabin-1* (*a*) and *nadrin-2* (*b*) in newly emerged queens and workers. The central panel illustrates the methylation patterns observed, with each column depicting a different methylation pattern, with red squares corresponding to methylated CpGs and grey squares to unmethylated CpGs. Barplots illustrate the estimated frequency of each pattern on the plus strand (above) and minus strand (below), averaged across three replicates.

In *cabin-1* and *nadrin-2*, a subset of patterns was found to be exclusive to each caste (figure 7). In *cabin-1*, caste-specific patterns were found to be rare. The most abundant patterns in each caste were observed in queens and workers (figure 7). In *nadrin-2*, however, caste-specific patterns were more abundant than in *cabin-1* (figure 7). In *cabin-1* and *nadrin-2*, each caste (queen and worker) had a different dominant pattern; while the dominant patterns were present in both castes, they were not the most abundant pattern for both castes. Interestingly, in *cabin-1*, queen-specific patterns showed less methylation than the common and worker-specific patterns (figure 7). This is consistent with the lower methylation levels observed in differentially methylated genes in queens relative to workers [12,22]. In a previous study of differentially methylated genes in larval heads and adult brains in queens and workers, *cabin-1* and *nadrin-2* were found to be differentially methylated only in older adult brains, but not in larval heads [12]. Our results suggest that both genes also show different brain methylation patterns in newly emerged queens and workers.

## 4. Discussion

We show that CpG methylation is not always symmetrical in the newly emerged honeybee adult brain methylomes. The presence of rare, consistently hemimethylated sites supports findings from earlier studies in other species, suggesting hemimethylation can occur at the same site in all DNA fragments in a sample [7,9,24,34]. In the honeybee brain, these sites correspond to less than 0.04% of methylated sites. Couldrey's study of the sheep muscle methylome has revealed a similarly low proportion of consistently hemimethylated sites, despite up to 10 times more methylated CpGs in total [7]. These hemimethylated sites in the honeybee, in addition to the sheep and wasp methylomes [24], while rare, may play a functional role. Although the specific function of these sites is unclear, one possibility is that they may reflect de-methylation and the general dynamics of methylation in neuronal cells in which the turnover of DNA methylation is more rapid than in other cell types [35].

The most compelling evidence for asymmetric methylation in the honeybee comes from the analysis of methylation patterns in amplicons within genes *cabin-1* and *nadrin-2*. While the original pairing of patterns on individual DNA fragments could not be deduced, the observed divergences in strand-specific pattern frequencies imply that some DNA fragments have a distinct methylation pattern on each strand. This result suggests that one or more of the CpG sites is hemimethylated. By identifying asymmetry in methylation patterns in our amplicons and detecting consistently hemimethylated sites, our study provides new evidence that rare asymmetrically methylated CpGs are present in brain methylomes. However, given the apparent scarcity of this type of methylation its functional relevance requires further investigation.

While the focus of this study was to investigate asymmetry, our analysis of methylation patterns also offers new insight into the heterogeneity of these patterns in brains of newly emerged honeybees. In the amplicons studied, a spectrum of methylation patterns were identified within replicates, suggesting that different individuals or cell types within the brain may have different methylation patterns (similar to those we have found in larval heads, or to inter-individual difference in gene-specific methylation in human brain) [15,36]. Interestingly, individual replicates within each category show differences in methylation patterns. Pooling of brains in each sample would be expected to normalize differences between individuals; however, our data do not support this expectation. Indeed, we find clear-cut differences between replicates, suggesting heterogeneity in methylation patterns within each category. Although up to 20–30 different methylation patterns were observed in *cabin-1* and *nadrin-2*, they correspond to a small fraction of all possible patterns. As each site can be methylated or unmethylated, the total number of possible patterns in a locus with *n* CpG sites is *2^n^*. In the gene *nadrin-2*, for example, we observed 30 out of 32 768 (2^15^) possible patterns. The relatively low number of patterns observed suggests that while some CpGs are variably methylated or unmethylated, others have a fixed methylation status. In *nadrin-2*, for example, three CpGs were consistently unmethylated, while the others were variable. CpGs may be variably methylated or unmethylated due to stochasticity of methylation mechanisms or due to single-nucleotide polymorphisms altering the sequence at a CpG, thereby preventing methylation of a site in some cells [17,32,33]. Our observation of a small but diverse spectrum of methylation patterns has several possible interpretations. If methylation of a single CpG can alter expression, as suggested by several studies [17,18,37–39], then the methylation status of each CpG in a pattern may be important. Each methylation pattern may result in slight differences in expression, suggesting that the epigenetic signal is fine-tuned by individual CpGs [17]. Alternatively, the general methylation pattern may be more important than methylation of individual CpGs [33]. Previous studies have suggested that the average methylation density of a region is maintained across cell divisions, rather than site-specific methylation [33,40,41]. Therefore, variations on the same general pattern may result in the same expression patterns and phenotype. That is, a certain level of noise and variability in methylation patterns may be tolerated. If this is the case, it would suggest that hemimethylation at some CpGs has little or no effect on function.

### 4.1. Queens and workers show differences in methylation

Our data also provide further evidence for differential methylation between queen and worker castes. For instance, asymmetry is manifested differently between castes; the majority of asymmetrically methylated CpGs detected were found to be caste-specific. This difference may reflect differences in the CpGs that are methylated in each caste [12,22]. Alternatively, asymmetrical sites may be methylated in both castes, but asymmetrical in only one. Another possibility is that the number of asymmetrically methylated CpGs observed in the queen dataset may be due to the higher coverage in the dataset, leading to increased sensitivity of methylation-level analysis. In addition, comparison of methylation patterns in queens and workers suggests that methylation patterns vary between castes. *cabin-1* and *nadrin-2* were found to have caste-specific patterns. The observation that queen-specific patterns were less methylated in *cabin-1* is consistent with the lower methylation levels observed in differentially methylated genes in queens relative to workers [12,22].

Importantly, differential methylation could be highly context-dependent. In our previous work using whole methylome approach, *cabin-1* and *nadrin-2* were found to be differentially methylated in older brains from queens and workers [22], but not in larval heads [12]. This study not only confirms that both genes are differentially methylated in brains of newly emerged queens and workers, but also suggests that such patterns may be tissue-dependent. Another possibility is that differential methylation was not seen previously in larval heads because the methylation levels mask underlying asymmetry in methylation patterns, thus obscuring differences between castes.

Differences in methylation patterns between castes, however, may not be biologically meaningful if small differences in methylation patterns do not translate to differences in expression or alternative splicing. The importance of the observed variation in methylation patterns and differences between castes is therefore dependent on whether different patterns correspond to functional differences.

Finally, the depth of our analyses combining bioinformatics and strand-specific deep amplicon sequencing not only contradicts a recent claim that virtually all cytosine methylation in this organism is asymmetrical [23], but also effectively rules out such a possibility.

## 5. Conclusion

Our findings challenge the assumption that symmetrical methylation levels reflect symmetry in the underlying methylation patterns. Differences in methylation levels reveal only consistent hemimethylation, whereas methylation patterns capture both consistent and random hemimethylation, as information from each read is retained rather than averaged. Consequently, these findings suggest that methylation levels are not a good predictor of methylation symmetry and that hemimethylation may occur more frequently than indicated by methylation levels.

## Supplementary Material

Figure S1

## Supplementary Material

Figure S2

## Supplementary Material

Figure S3

## Supplementary Material

Figure S4

## Supplementary Material

Figure S5

## Supplementary Material

Figure S6

## Data accessibility

The datasets supporting this article have been uploaded as part of the electronic supplementary material and at the Dryad Digital Repository (http://dx.doi.org/10.5061/dryad.7nb8q) [28].
