# Supplementary figures and images for: Detecting rare asymmetrically methylated cytosines and decoding methylation patterns in the honeybee genome
Source: R Soc Open Sci. 2017 Sep 6;4(9):170248. doi: 10.1098/rsos.170248 (PMC5627074; doi:10.1098/rsos.170248)

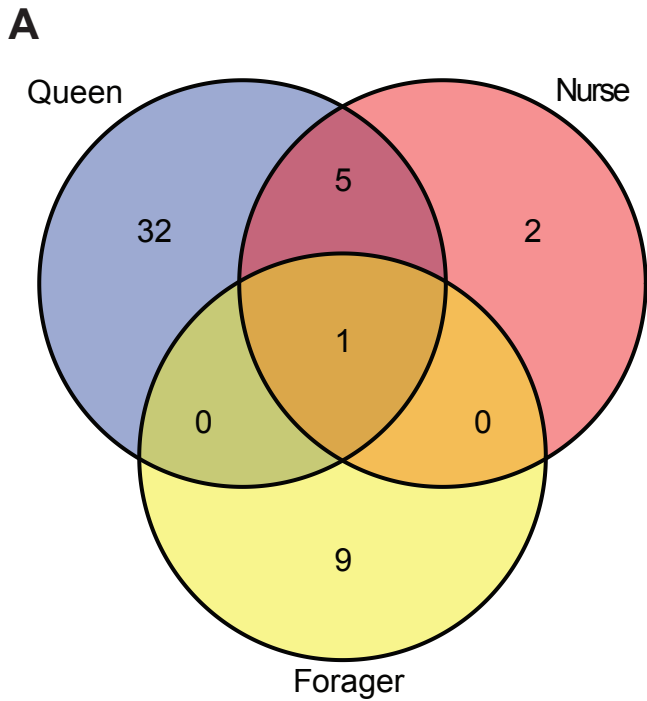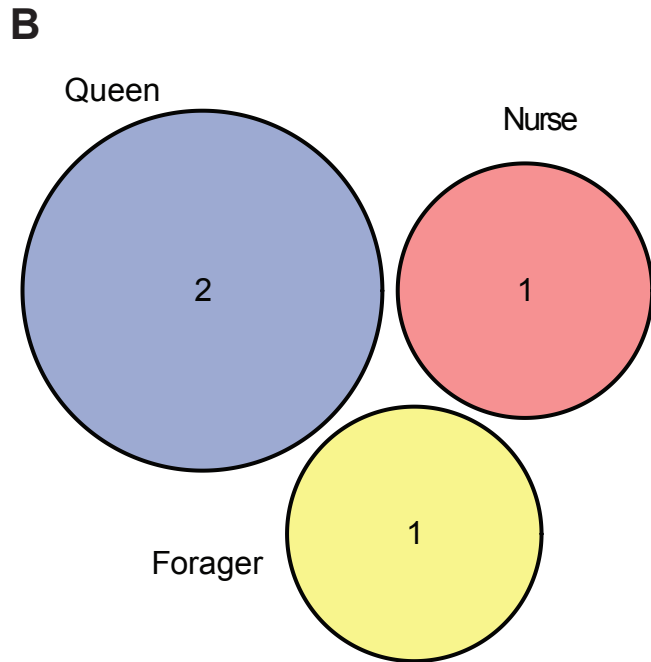

Supplement: Figure S1 [file rsos170248supp1.pdf]

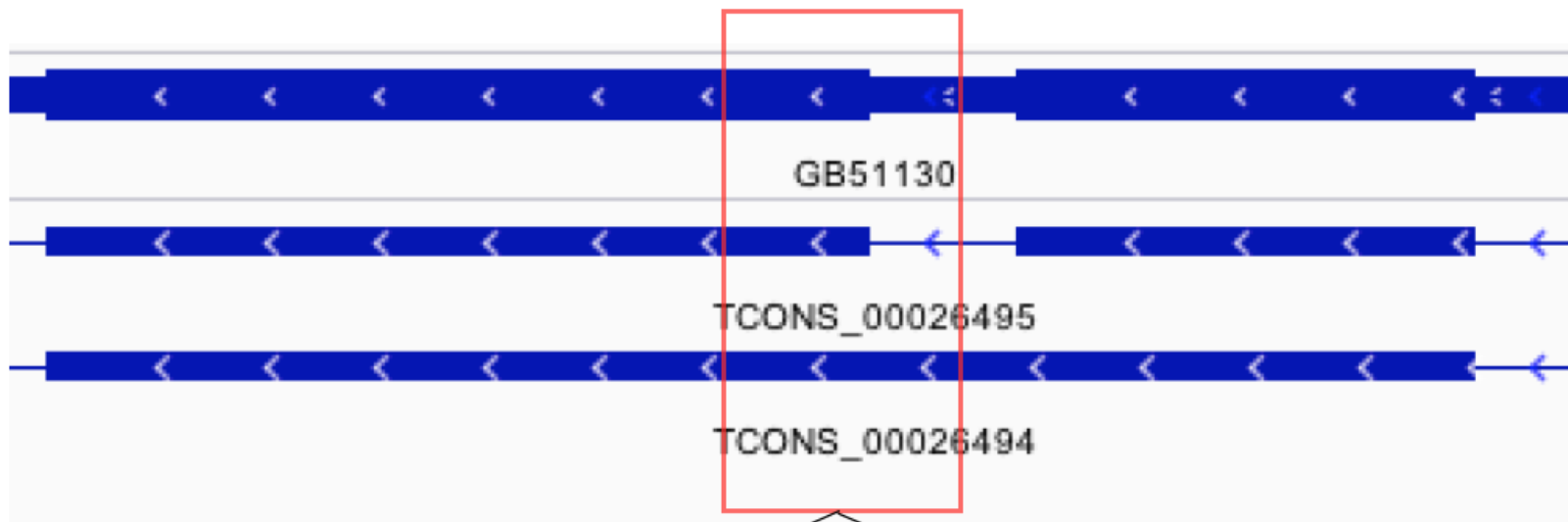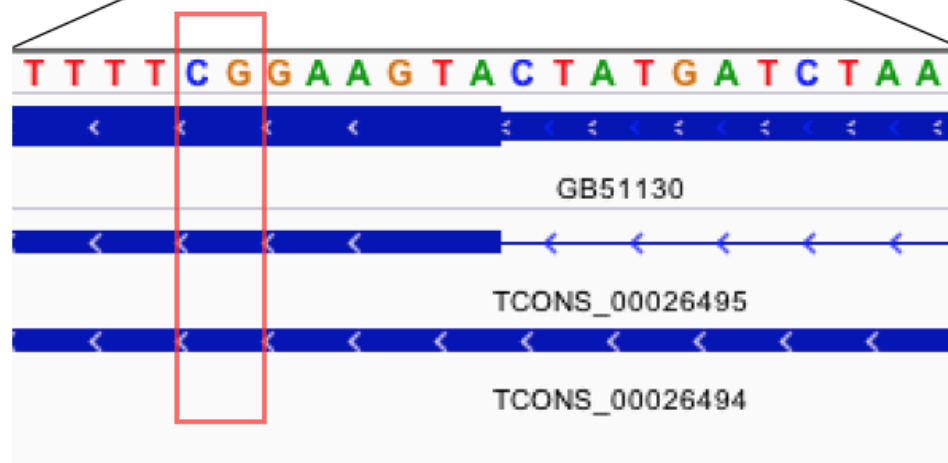

Supplement: Figure S2 [file rsos170248supp2.pdf]

Queen

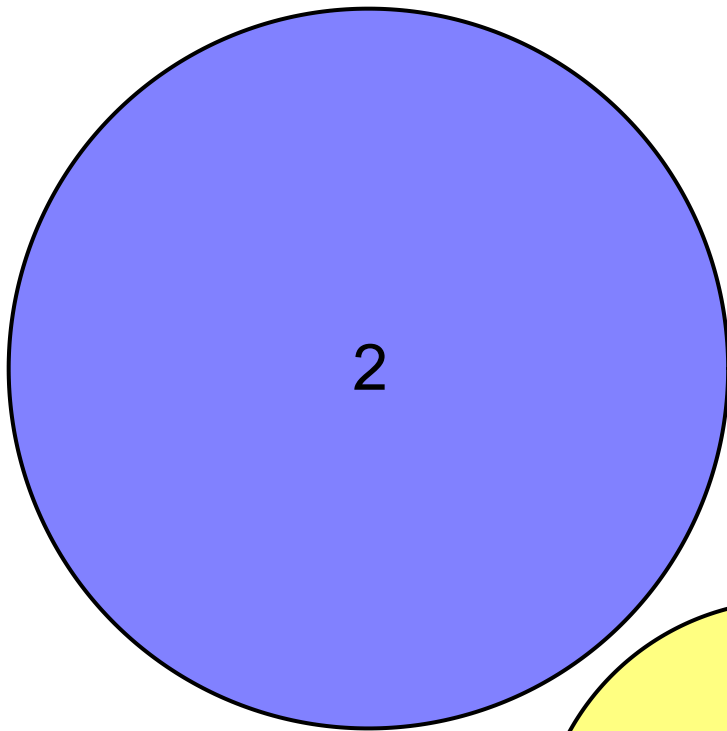

Nurse

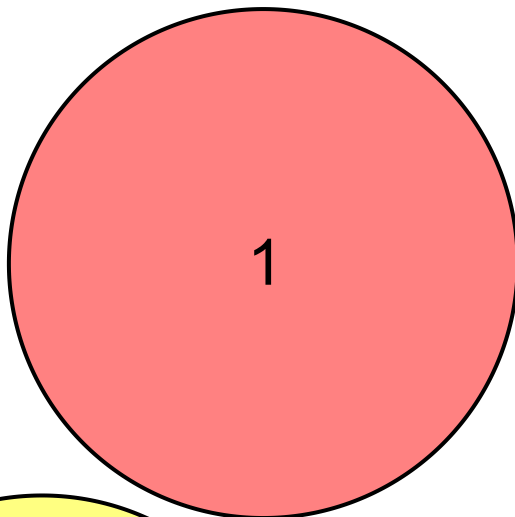

Forager

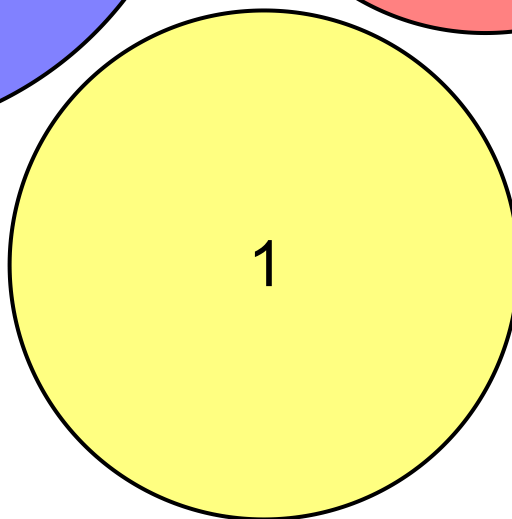

Supplement: Figure S3 [file rsos170248supp3.pdf]

Difference in proportions of patterns between strands (Plus - Minus strand)

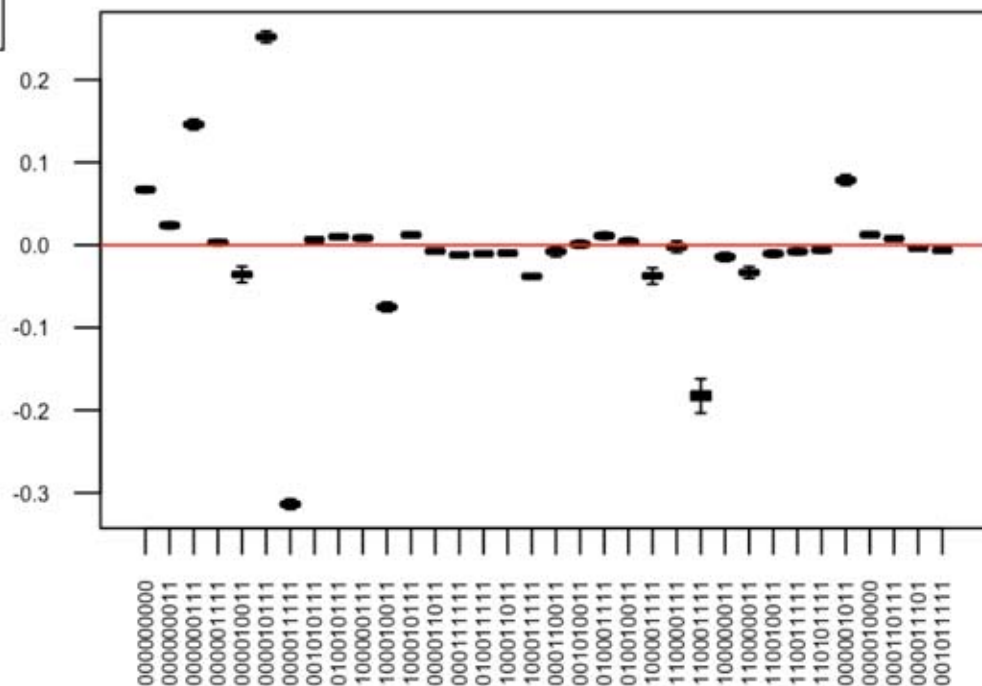

# B

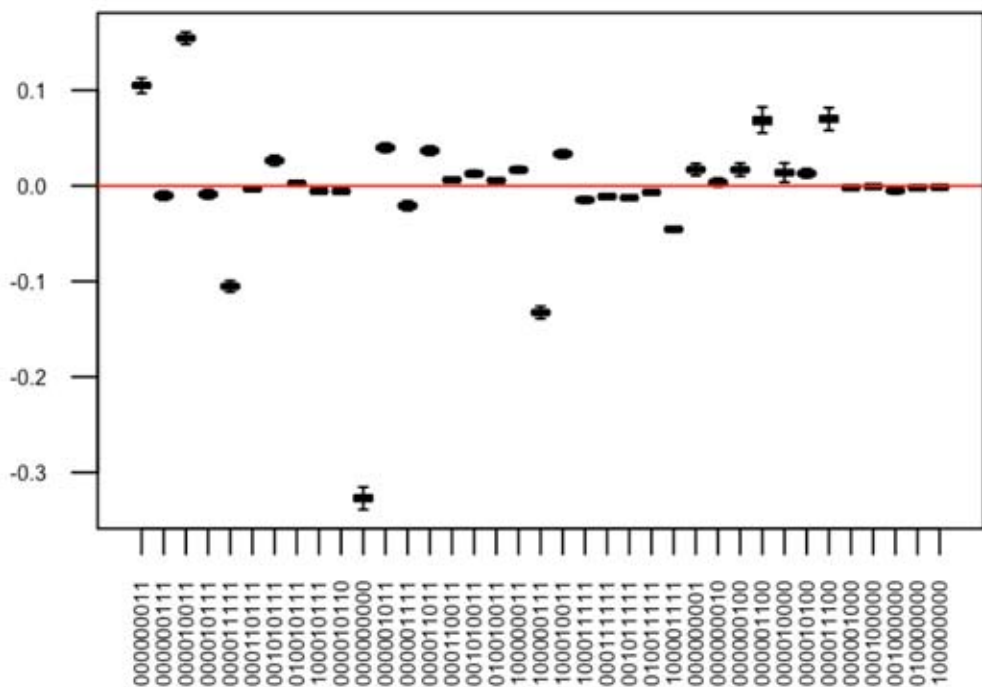

Supplement: Figure S4 [file rsos170248supp4.pdf]

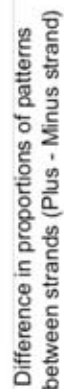

**B**

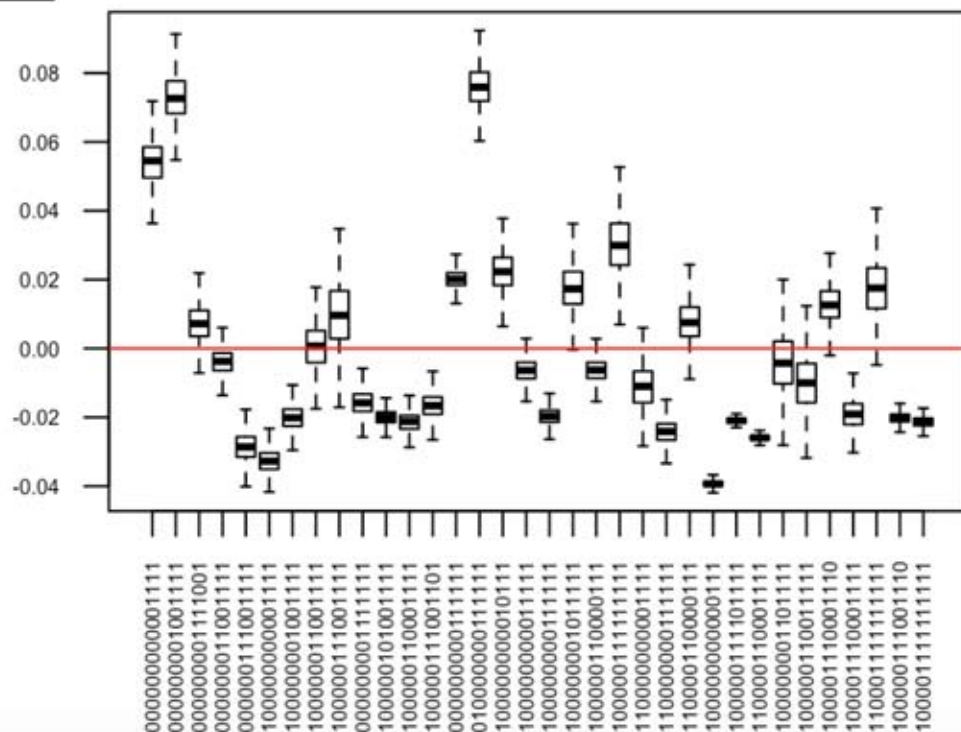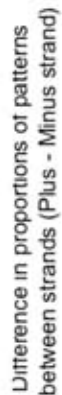

# A

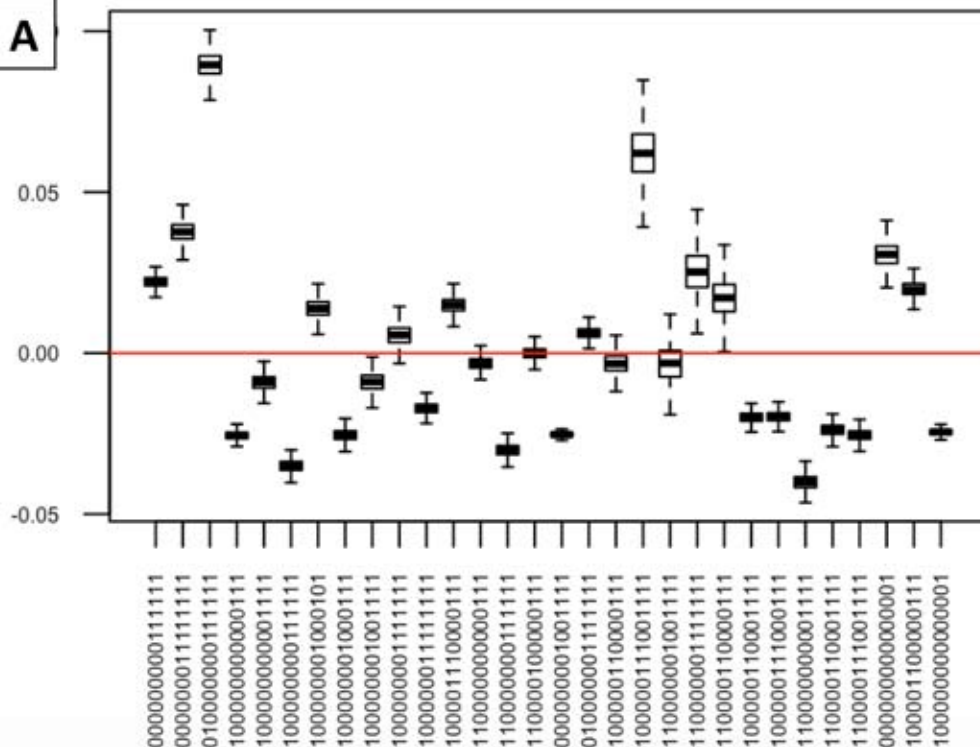

Supplement: Figure S5 [file rsos170248supp5.pdf]
