# Supplementary material for: Detecting rare asymmetrically methylated cytosines and decoding methylation patterns in the honeybee genome
Source: R Soc Open Sci. 2017 Sep 6;4(9):170248. doi: 10.1098/rsos.170248 (PMC5627074; doi:10.1098/rsos.170248)

**Figure S6.** Comparison of patterns in at QWFC1 and flanking CpGs in newly-emerged queens and workers. The central panel illustrates the methylation patterns observed, with each column depicting a different methylation pattern, with red squares corresponding to methylated CpGs and grey squares to unmethylated CpGs. Barplots illustrate the estimated frequency of each pattern on the plus strand (above) and minus strand (below), averaged across three replicates.


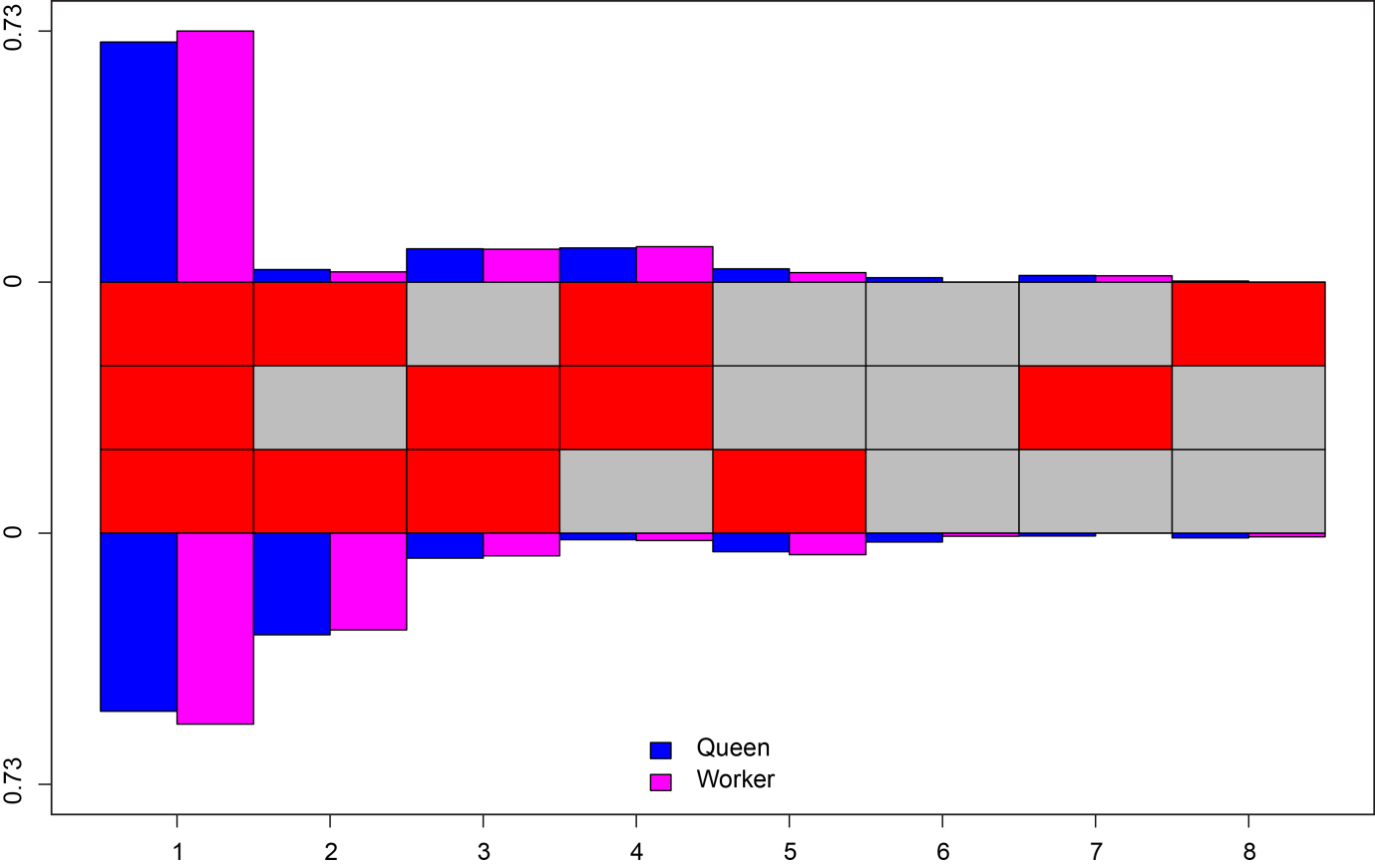

Supplement: Figure S6 [file rsos170248supp6.docx]
